# Additional Value of PET and CT Image-Based Features in the Detection of Occult Lymph Node Metastases in Lung Cancer: A Systematic Review of the Literature

**DOI:** 10.3390/diagnostics13132153

**Published:** 2023-06-23

**Authors:** Priscilla Guglielmo, Francesca Marturano, Andrea Bettinelli, Matteo Sepulcri, Giulia Pasello, Michele Gregianin, Marta Paiusco, Laura Evangelista

**Affiliations:** 1 Nuclear Medicine Unit, Veneto Institute of Oncology IOV—IRCCS, 35128 Padua, Italy; 2 Medical Physics Unit, Veneto Institute of Oncology IOV—IRCCS, 35128 Padua, Italy; 3 Radiotherapy, Veneto Institute of Oncology IOV—IRCCS, 35128 Padua, Italy; 4 Department of Surgery, Oncology and Gastroenterology, University of Padua, 35128 Padua, Italy; 5 Medical Oncology 2, Veneto Institute of Oncology IOV—IRCCS, 35128 Padua, Italy; 6 Nuclear Medicine Unit, Department of Medicine DIMED, University of Padua, 35128 Padua, Italy

**Keywords:** lung cancer, radiomics, deep learning, occult lymph node metastasis, CT, PET/CT, FDG

## Abstract

Lung cancer represents the second most common malignancy worldwide and lymph node (LN) involvement serves as a crucial prognostic factor for tailoring treatment approaches. Invasive methods, such as mediastinoscopy and endobronchial ultrasound-guided transbronchial needle aspiration (EBUS-TBNA), are employed for preoperative LN staging. Among the preoperative non-invasive diagnostic methods, computed tomography (CT) and, recently, positron emission tomography (PET)/CT with fluorine-18-fludeoxyglucose ([^18^F]FDG) are routinely recommended by several guidelines; however, they can both miss pathologically proven LN metastases, with an incidence up to 26% for patients staged with [^18^F]FDG PET/CT. These undetected metastases, known as occult LN metastases (OLMs), are usually cases of micro-metastasis or small LN metastasis (shortest radius below 10 mm). Hence, it is crucial to find novel approaches to increase their discovery rate. Radiomics is an emerging field that seeks to uncover and quantify the concealed information present in biomedical images by utilising machine or deep learning approaches. The extracted features can be integrated into predictive models, as numerous reports have emphasised their usefulness in the staging of lung cancer. However, there is a paucity of studies examining the detection of OLMs using quantitative features derived from images. Hence, the objective of this review was to investigate the potential application of PET- and/or CT-derived quantitative radiomic features for the identification of OLMs.

## 1. Introduction

Lung cancer is one of the most commonly diagnosed malignancies and a leading cause of cancer-related deaths worldwide, with an estimated 1.8 million deaths per year [1]. In particular, non-small cell lung cancer (NSCLC) accounts for approximately 85% of all cases [2]. Among the subtypes of NSCLC, the most prevalent is adenocarcinoma [3].

The standard treatment for early-stage lung cancer patients is surgery, which includes anatomical resection and systematic nodal dissection. This approach results in a 70% survival rate for patients over 5 years, despite a recurrence rate of 55–75%. Moreover, limited surgery options, such as wedge resection or sublobar resection, are viable for lung cancer patients without lymph node (LN) metastases, making it possible to preserve healthy lung tissue and, consequently, spare lung function [4,5,6]. Conversely, for patients with potentially resectable mediastinal LN metastases, radical resection is recommended. In cases where surgery is not possible, inoperable patients are treated with stereotactic radiotherapy, which offers a 3-year survival rate close to 60% [7].

LN involvement has been proven to be an important prognostic factor for NSCLC and plays a role in tailoring its treatment [8]. The International Association for the Study of Lung Cancer (IASLC) has suggested that the clinical and pathological LN statuses are closely associated with the 5-year survival rate [9]. Therefore, LN staging is a crucial step in the early-stage detection of lung cancer.

Currently, mediastinoscopy and endobronchial ultrasound-guided transbronchial needle aspiration (EBUS-TBNA) are considered the gold standard for preoperative LN staging, but they are not routinely recommended due to their invasiveness [10,11,12,13]. Among preoperative non-invasive diagnostic methods, computed tomography (CT) is deemed the standard imaging tool for lung cancer, as it provides detailed information about tumour location, size, and spread [14]. For CT, the criterion for diagnosing LN involvement is when the shortest axis of the LN is greater than 10 mm [15]. In recent decades, positron emission tomography (PET)/CT with fluorine-18-fludeoxyglucose ([^18^F]FDG) has been extensively used in the preoperative setting for patients with lung cancer. This imaging approach is considered a key non-invasive staging method [16] and has shown better performance in LN staging (estimated sensitivity of 77% and specificity of 86%) compared to CT alone (55% and 81%, respectively) [17,18,19]. For PET imaging, the criterion for diagnosing LN involvement is the presence of LN uptake above the intensity of the surrounding background activity, with or without LN enlargement in co-registered CT imaging.

Unfortunately, in early-stage NSCLC patients, the risk of locoregional recurrence due to missed detection of occult LN metastases (OLMs) is 26% [7]. OLMs refer to hilar and/or mediastinal LNs that appear negative in PET and/or CT imaging (clinical N0—cN0) due to the limited spatial resolution of these modalities [7] but are later identified as metastatic based on pathological assessment after surgery [20,21,22,23]. Typically, OLMs consist of micro-metastases composed of tumour-cell clusters ranging in size from 0.2 to 2.0 mm, depending on the size of the metastatic tumour cell [24]. The incidence of OLMs in PET/CT is estimated to be between 12.6 and 26.7% [20,21,22,23], whereas there are no available data for CT imaging. Inflammatory diseases, such as lymphadenitis or tuberculosis, can also lead to false-positive findings in both [^18^F]FDG PET and CT imaging [7], further highlighting the need to develop novel approaches for increasing the discovery rate of OLMs.

Currently, the identification of OLMs in lung cancer relies on clinical and radiological features, including lesion location (central vs. peripheral site), histology (adenocarcinoma vs. others) [25,26,27], and gender (male vs. female). Additionally, the metabolic tumour volume (MTV), total lesion glycolysis (TLG), maximum standardised uptake value (SUVmax) of the primary lesion [28], and tumour size derived from CT images have been considered to improve identification performance. MTV and TLG, which reflect metabolic information and tumour generation status, have long been regarded as good independent predictors of OLMs [29,30]. Moreover, an SUVmax for the primary tumour higher than 4–6 and a tumour size larger than 3 cm have been found to be associated with OLMs with an odds ratio ranging between 2.2 and 4.18 [27,31,32,33,34,35,36]. Therefore, the integration of CT and PET data holds promise for enhancing prediction performance for OLMs.

However, the variability in the reported cut-off values and the limited reproducibility of the abovementioned studies emphasise the need for the development of new models, thereby paving the way for novel approaches.

In recent years, it has become widely acknowledged that the information obtained from medical images using the naked eye is a limited representation and that a significant amount of valuable data remain concealed within the images [37]. Radiomics is a non-invasive approach typically applied to radiological images that makes it possible to capture and quantitatively describe several characteristics of a region of interest (ROI), including its morphology, intensity, and texture. These characteristics, either alone or in combination with other clinical or histopathological parameters, can serve as predictors for various clinical endpoints [37,38,39,40]. Traditional radiomic features based on standardised mathematical formulations are referred to as ”handcrafted” features. Additionally, thousands of ”deep” features can be extracted using deep learning models, which do not rely on an explicit mathematical formulation of the characteristic of interest. Numerous reports have highlighted the utility of handcrafted and deep features for the diagnosis, staging, and prognosis of lung cancer [41,42,43,44,45,46,47].

At present, there is a paucity of radiomic studies specifically focusing on the identification of OLMs in the setting of NSCLC, with the majority of studies based on CT radiomics [7,48]. A recent study used the texture parameters of [^18^F]FDG PET images combined with metabolic parameters (e.g., MTV) and serological data (e.g., carcinoembryonic antigen - CEA) to develop a radiomic nomogram that demonstrated good prediction results [3].

The aim of our study was to conduct a systematic review of the literature, providing an overview of the potential application, in terms of additional value over morphological and functional imaging alone, of PET and/or CT radiomic analysis in the detection of OLMs.

## 2. Materials and Methods

The systematic review was conducted in accordance with the Preferred Reporting Items for Systematic Reviews and Meta-Analysis (PRISMA) guidelines by P.G., F.M., A.B. and L.E. The authors conducted a search to identify prospective or retrospective studies that utilised radiomic analysis of CT and/or PET images for assessing OLMs. The most relevant databases and Web sources were searched using the following query: “(“lung adenocarcinoma” OR “lung cancer”) AND (“radiomics” OR “radiomic” OR “deep learning”) AND (“PET” OR “CT” OR “PET/CT”) AND (“occult lymph node metastasis” OR “OLM”)”. Only original articles in English published before October 2022 were considered.

After removing duplicates and excluding papers not relevant to the topic and review articles, the titles and abstracts of the retrieved records were carefully examined. The studies were selected based on the following criteria: (a) PET or CT data were used for radiomic analysis and (b) PET or CT examination had to be performed at the time of initial staging. The references cited in the selected articles were also screened to identify additional relevant studies.

To assess the quality of reporting in the radiomic studies, we computed the radiomic quality score (RQS) metric proposed by Lambin and colleagues [37]. The RQS ranges from 0 to a maximum of 36 points (100%) and evaluates 16 aspects related to (a) protocol quality and stability in image segmentation, (b) feature selection and validation, (c) biological/clinical validation and utility, (d) the model performance index, (e) the level of evidence, and (f) open science and data. To ensure a robust calculation of the RQS, two of the authors computed it independently and any discrepancies were resolved through discussion.

## 3. Results

**Figure 1:**
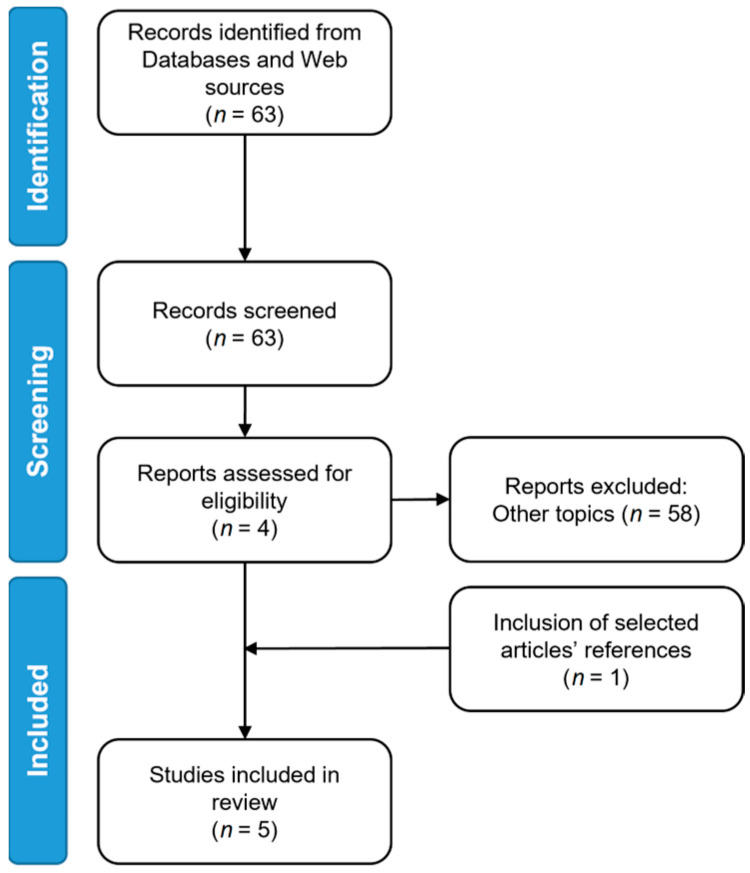
PRISMA flow diagram.

A total of 63 studies were screened for eligibility and 5 of them met the inclusion criteria for our review and were selected for further analysis (Figure 1). Among the five studies, four were based on handcrafted radiomic features, while the remaining study considered only deep features.

### 3.1. Study Populations

All studies were based on human subjects, with an average sample size of 354 patients (range: 228–492). The clinical characteristics of the selected articles are listed in Table 1. In all cases, the ground-truth assessment of the OLMs was undertaken via histopathological analysis. Qiao et al. [49] and Ouyang et al. [50] included only patients with lung adenocarcinoma, while Wang et al. [3] considered patients affected by either adenocarcinoma or squamous cell cancer.

### 3.2. The Workflow of Radiomic Analysis

**Figure 2:**
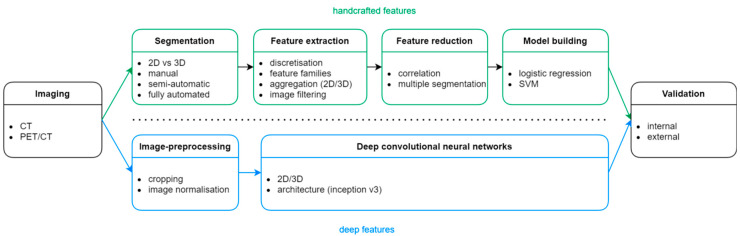
The workflow for machine learning-based radiomic studies (top) and deep-learning ones (bottom).

Despite the diversity found in the literature, radiomic studies generally follow a conceptually straightforward workflow consisting of a series of well-defined, conventional steps (Figure 2).

Machine learning-based radiomic studies that rely on handcrafted radiomic features typically involve the following key steps: image acquisition, feature extraction, feature selection, model development, and, ultimately, model validation.

In contrast, deep learning-based radiomic studies typically have a streamlined workflow that includes fewer steps, such as image acquisition, image pre-processing, neural network training, and model validation. In this case, the segmentation step is not necessary and both feature extraction and selection are embedded within the process of training the artificial neural network.

In this review, we present and compare the five selected original papers in relation to the aforementioned workflow. A comprehensive summary of the methodologies and results from these studies can be found in Table 2.

#### 3.2.1. Imaging Acquisition Protocol

Two out of the five studies (i.e., those by Zhong et al. [48] and Zhang et al. [7]) performed the radiomic analysis exclusively with CT imaging. On the other hand, the studies by Qiao et al. [49] and Ouyang et al. [50] considered both CT and PET data. However, there were differences in the imaging protocols used: Zhong et al. [48] analysed unenhanced chest CT scans acquired with a 1 mm slice thickness with 64–128 multidetector computed tomography (MDCT) scanners, while Zhang et al. [7] resorted to the venous phase of contrast-enhanced CT imaging acquired with a 5 mm slice thickness with a 64-MDCT scanner. Qiao et al. [49] and Ouyang et al. [50] acquired the CT imaging with hybrid PET/CT systems and slice thicknesses of 3.75 mm and 5 mm, respectively.

The study by Wang et al. [3] instead focused exclusively on PET imaging, which was acquired in supine position 60 min after an intravenous injection of 3.7 MBq/kg of [^18^F]FDG. The PET images were reconstructed with a time-of-flight algorithm and a voxel size of 4 × 4 × 5 mm. Consistent with the parameters used by Ouyang et al. [50], Qiao et al. [49] acquired the PET imaging 60 min after the injection of 3.70–5.55 MBq/kg of [^18^F]FDG and used a 3D-OSEM reconstruction algorithm with an unknown voxel size.

#### 3.2.2. ROI Segmentation

ROI segmentation is a crucial step in the radiomic workflow as it determines the specific voxels where handcrafted features will be computed. The process of segmentation can be carried out manually by expert physicians using various techniques, such as drawing polygons to define the ROI enclosures on a slice-by-slice basis or employing assisting tools, such as 2D or 3D adaptive brushes [51]. Alternatively, segmentations can be performed in a semi-automatic manner, utilising methods such as region-growing [52] or thresholding [53], although manual correction is often required. Complete automation of segmentation is also possible by employing architectures such as U-Net [54].

Although manual segmentations are considered the gold standard, they have several drawbacks. Firstly, depending on the type of segmentation (2D versus 3D), they can be time-consuming. Secondly, they are vulnerable to both inter-reader and intra-reader variability, which can introduce inconsistencies in the results if not appropriately addressed and accounted for.

In the specific context of this review, due to the nature of OLMs being inherently invisible in imaging, direct segmentation of the lymph node itself is not feasible. Additionally, the limited volume of lymph nodes poses a challenge for textural quantification as, from a statistical perspective, the restricted number of voxels within the segmentation does not allow robust and meaningful characterization of the ROI. As a result, all the studies segmented or analysed the primary tumour site instead. In the four studies based on handcrafted radiomic features [3,7,48,49], segmentations were performed manually and, in one case, integrated with semi-automatic methods. Specifically, in the studies conducted by Zhong [48], Zhang [7], and Wang et al. [3], multiple segmentations were also obtained by a second independent expert clinician. Multiple segmentations allowed the implementation of a preliminary feature reduction step based on the feature variability across readers, effectively discharging features that were unstable because highly sensitive to small segmentation differences.

Conversely, in the study conducted by Ouyang [50], which exclusively explored deep features, the segmentation of the region of interest (ROI) was not required and only square cropping around the chest area was performed.

#### 3.2.3. Feature Extraction

All four studies that used handcrafted radiomic features flanked first-order features, including statistics descriptors of the ROI, with textural features; namely, the grey-level co-occurrence matrix (GLCM) [55] and the grey-level run-length matrix (GLRLM) [56]. Three studies also included morphological features—the grey-level size zone matrix (GLSZM) [57] and the neighbouring grey-tone difference matrix (NGTDM) [58] families—while only two studies included the neighbouring grey-level dependence matrix (NGLDM) [59]. More details about these feature families can be found in the IBSI manual [60].

Prior to feature extraction, image filtering [61] was also applied to enhance specific image characteristics. Three studies employed wavelet-based filtering methods, but local binary pattern and Laplacian of Gaussian filtering were also used.

Different software programs were employed for feature extraction across the studies. In particular, Mazda [62], PyRadiomics [63], Region Studio (Regiontec Ltd., Shanghai, China), and the Artificial Intelligence Kit (A.K, version 3.2.0, GE Healthcare) were used.

Ultimately, Ouyang et al. [50] employed a highly efficient deep neural network, Inception v3 [64], which was fed with three 2D slices (axial, coronal, and sagittal) cropped around the primary lesion. The convolutional layers’ weights were pretrained on the ImageNet dataset [65], while only the last classification layers were fine-tuned for the task of OLM prediction.

#### 3.2.4. Feature Selection and Machine Learning Models

A common challenge in the development of AI models is the presence of a higher number of features (or predictors) compared to the number of samples (e.g., the subjects of the study). This imbalance can lead to overfitting, potentially reducing the model’s performance and hindering its ability to generalise to unseen data. One approach to address this issue is to reduce the number of features by considering feature inter-correlation, repeatability, and reproducibility [66].

All three studies that evaluated multiple segmentations (i.e., those by Zhong [48], Zhang [7], and Wang [3]) used the intraclass correlation coefficient (ICC) to assess the inter-reader reproducibility of radiomic features. Wang et al. [3] additionally examined the intra-reader reproducibility by analysing repeated segmentations performed by the same reader with a one-week interval. The threshold for considering a feature as reproducible ranged from 0.75 to 0.90. In addition to or as a replacement of ICC, other feature reduction methods were used, such as hierarchic clustering analysis, principal component analysis, the least absolute shrinkage and selection operator (LASSO), extremely randomised trees, backward selection, and univariate logistic regression.

After the feature selection step, Zhang [7], Wang [3], and Qiao [49] constructed radiomic scores based solely on the radiomic features extracted from the ROI using multivariable logistic regression. Subsequently, they integrated the scores with clinical variables to build nomograms, resulting in improved prediction performance compared to the radiomic scores alone.

On the other hand, Zhong et al. [48] obtained the radiomic signature using support vector machines, where the hyperparameters were optimised through a 10-fold cross-validation procedure. The model with the best hyperparameters was finally evaluated using a 5-fold stratified cross-validation with 100 repetitions. The performance of the radiomic signature was then compared to the clinical histopathologic model.

#### 3.2.5. Deep-Learning Models

Deep learning is a discipline that employs artificial neural networks to autonomously learn feature representations from images. This field of study has brought about revolutionary advancements in several domains, demonstrating its capability to surpass human-level performance in specific tasks [67]. Deep learning architectures offer solutions to current challenges in image analysis, including image segmentation (e.g., U-Net [54]), feature extraction (e.g., autoencoders [68]), and classification. These architectures enable comprehensive image evaluation and autonomous extraction of relevant information, eliminating the need for manual delineations.

Convolutional neural networks, such as the residual network [69], EfficientNet [70], DenseNet [71], and Inception [64], have been widely proposed for image analysis. More recently, transformer-based models, including vision transformers [72], have attracted interest due to their ability to capture long-range dependencies and contextual information.

In our literature review, we identified only one study (by Ouyang and colleagues [50]) that applied deep learning methods for OLM prediction. The authors developed three distinct deep learning models based on the Inception v3 network, which is designed to extract relevant features at various scales and resolutions using multiple parallel convolutional layers. One model was specifically designed for CT imaging, another for PET imaging, and a third model for the integration of both imaging modalities. The deep learning model showed promising predictive performance in identifying patients suitable for limited resection. However, the authors acknowledged the black box nature of their model as one of the main limitations, despite the encouraging results.

### 3.3. Model Results and Additional Value of Radiomics over Clinical Information

For the models based on handcrafted radiomic features (namely, the studies by Zhang [7], Wang [3], and Qiao [49]), the authors built nomograms to predict OLMs by incorporating the radiomic score and clinical variables. These variables included the CT-reported tumour size, T stage, tumour type, and CEA for Zhang (AUC of 0.81 with the validation cohort); the CEA and MTV for Wang (concordance index = 0.77 with the validation cohort); and the tumour location for Qiao (AUC of 0.88 in the testing set). However, only Zhang et al. [7] found that tumour size and radiomic score were independent predictors of OLMs in multivariate analysis.

On the other hand, Zhong et al. [48] obtained the radiomic signature from the SVM model and then compared it to the clinical histopathologic model (based on age, sex, tumour location, tumour diameter, and histological subtype). The best SVM model trained to distinguish OLMs based on the radiomic signature achieved an AUC, accuracy, sensitivity, and specificity of 0.97, 0.91, 0.95, and 0.92, respectively. The greater effectiveness of the radiomic signature compared to the clinical histopathologic model was proved using a multivariable logistic regression model. The radiomic signature alone achieved an accuracy of 0.81, while the clinical data alone achieved 0.61 accuracy.

The deep learning models used by Ouyang et al. [50] achieved AUCs of 0.79, 0.73, and 0.87 for CT only, PET only, and the combined imaging, respectively, with a prospective test set. Their results showed that the complex model (i.e., [^18^F]FDG PET and low-dose CT concatenated with fully connected and sigmoid layers) provided the best diagnostic performance in identifying patients with OLMs.

## 4. Discussion

There is growing evidence supporting the use of stratification tools that combine clinical parameters, genomic biomarkers, and morphological and functional features to predict OLMs in lung cancer [73] and potentially optimise healthcare.

The urgent need to develop a more effective method for preoperatively predicting OLMs has inspired numerous studies, which have resorted to high-throughput image analysis approaches, such as radiomic approaches, to extract quantitative image-based features that provide information about the underlying tumour biology and behaviour [37,74].

Several studies have investigated the value of radiomic machine learning or deep learning approaches in relation to different aspects of NSCLC [42,75,76,77], but few studies have focused on the detection of OLMs. These studies are primarily based on CT-radiomics [7,48], while only 1% of the radiomic studies were performed in the field of nuclear medicine [78].

Prediction models proposed in the last decade [79] were established mainly by analysing the texture of the primary tumour to predict LN involvement, as LNs are typically too small to be analysed through image-related approaches or may be occult in preoperative PET images. The integration of radiomic and clinical data contributed to the development of classification nomograms for OLMs, which have shown good predictive accuracy in patients with lung adenocarcinoma.

A common aspect found in the studies analysed in this review was that they all identified multiple radiomic feature-based signatures derived from the primary tumour. These signatures demonstrate the potential to predict OLMs better than clinicohistopathological features alone and have shown incremental value in the preoperative prediction of pathological LN status, delineating a scenario where radiomics aids decision making. Indeed, the AUCs for predicting OLMs by using radiomic models ranged from 0.87 to 0.97 compared to 0.72 to 0.81 for the clinical models.

### 4.1. Limitations

Despite the promising results, we ought to discuss the limitations that we have identified in these studies.

#### 4.1.1. Clinical Aspects

The five selected studies only included patients with adenocarcinoma or squamous cell carcinoma, while other histological types and lesions that exhibited ground-glass density were not considered. These factors contribute to hindering the application of their results in clinical routines.

None of the studies stated whether LNs were removed through a complete and rigorous mediastinal dissection.

Additionally, the study by Ouyang et al. [50] excluded patients with multifocal lung cancer due to the difficulty of determining which lesion would have caused the occult lymph node metastases.

#### 4.1.2. Technical Aspects

Image acquisition and reconstruction parameters play a role in the reproducibility of these studies. For example, voxel size can impact features that depend on voxel volume [80] and, even if interpolation can be used to harmonise it, the frequency content contained within the interpolated images might still reflect the original voxel size (e.g., slice thickness of 5 versus 1 mm). He et al. [81] demonstrated that reconstruction slice thickness and convolution kernel can affect the performance of radiomic signatures in pulmonary nodules, suggesting that these factors should be considered when collecting patients for external validations.

Moreover, the variety of the considered feature families, along with the lack of reporting regarding specific feature-extraction parameters, renders the studies difficult to compare and reproduce.

To address these technical issues, one potential approach is to utilise publicly available protocols for data acquisition and implement data harmonisation methods, such as ComBat [82]. Additionally, it is crucial to closely adhere to the IBSI recommendations [60] regarding image pre-processing steps, feature calculation, and reporting.

Regarding deep learning, the application of such models in medicine poses several challenges that need to be addressed. These include the requirement for large amounts of data, the high computational costs for training complex models, and the need for result interpretability. Transfer learning techniques offer a practical solution to mitigate the requirement for extensive datasets: by leveraging pretrained models and selectively fine-tuning specific parameters for the task at hand, transfer learning allows the application of such models in the medical research field, particularly in scenarios where data availability is limited. Concurrently, saliency maps and activation maps fulfil the need for model interpretability, providing valuable insights into the inner workings of the model.

#### 4.1.3. Sample Imbalance

Occult lymph node metastases are a challenging setting as, by definition, they elude diagnostic criteria. In general, the percentage of OLMs is low compared to the detected cases, which is considered favourable from a clinical standpoint. However, it is unfavourable for classification modelling. Class imbalance poses a challenge as most machine learning algorithms assume an equal number of samples for each class, and this may lead to poor predictive performance for the minority class, which is usually the one of interest.

In the selected studies, the percentages of OLMs ranged between 18% and 41%, raising concerns for model training. However, none of the studies based on handcrafted features employed class-balancing techniques (e.g., over/undersampling methods, such as the synthetic minority oversampling technique (SMOTE) [83] or adaptive synthetic sampling (ADASYN) [84]), only reporting class imbalance as a major limitation. In contrast, Ouyang et al. [50] did employ oversampling to address this issue, albeit in a non-conventional manner, applying it to both the minority and majority classes.

While it is advisable to apply class-balancing techniques when necessary, selecting a specific technique over others is non-trivial due to their respective drawbacks. For example, oversampling the minority class can lead to overfitting, while undersampling the majority class may result in the loss of crucial information.

#### 4.1.4. External Validation and Sample Size

Four out of five studies were single-centre retrospective studies with a reported small sample size, potentially leading to data selection bias. External validation was absent in these studies, except for the one by Zhang et al. [7]; however, the validation sample size was modest and the design remained retrospective.

To validate the accuracy of the proposed nomograms before clinical translation, it is recommended to utilise large datasets with prospectively enrolled patients imaged with common public acquisition protocols. Another option to consider is the use of data augmentation approaches, which enable the expansion of the dataset by generating synthetic images. However, common data augmentation techniques, such as image rotation and scaling, are not commonly employed in radiomics based on handcrafted features: these techniques are primarily used to assess feature reproducibility [85] rather than to generate new data. Alternatively, deep learning approaches, such as generative adversarial networks [86], can be used to generate synthetic data. Nevertheless, it should be noted that these approaches often require a substantial amount of data themselves, which may limit their applicability as data augmentation methods.

Multi-centre studies and external validation datasets are crucial for establishing robust research results and thereby facilitating the advancement of this research field. Future studies should also explore the integration of their datasets with publicly available data. Nevertheless, to the best of our knowledge, there are currently no publicly available datasets within repositories such as The Cancer Imaging Archive (TCIA) that precisely align with the research focus of predicting OLMs. Furthermore, none of the reviewed studies adhered to the principles of findable, accessible, interoperable, and reusable (FAIR) data sharing [87].

### 4.2. Future Perspectives

Based on our perspective and the findings of this literature review, the initial step should involve external validation of the nomograms proposed by Qiao [49], Wang [3], and Zhang [7], as they provide enough information to replicate their studies. Criteria for publication should prioritise the overall quality of the study (e.g., the RQS) and the level of evidence, regardless of the validation outcome. Negative validation results could challenge the proposed models and encourage investigators to delve deeper into confounding factors, while positive outcomes would bring these models closer to clinical implementation.

Future studies should also explore more holistic models that encompass various factors, such as demographic information (e.g., gender, age, ethnic origin, geographical location), personal habits (e.g., smoking, occupational exposure), pre-existent clinical conditions (e.g., diabetes, obesity, chronic obstructive pulmonary disease), genetic features (e.g., family history, gene expression, genetic alterations), tumour biology (e.g., histopathology, immunohistochemistry analysis, marker expression), and radiomics. Prospective randomised clinical trials, methodological standardisation, data sharing, and software accessibility are additional important considerations to enhance the applicability and reusability of published studies [37].

Furthermore, it is crucial for forthcoming investigations to prioritise the interpretability of machine learning and deep learning models. Techniques such as feature importance analysis can provide insights into the outcomes of machine learning models, while methods such as saliency maps can assist in comprehending the results generated by deep learning models. Additionally, the utilisation of effective data visualisation approaches can also play a pivotal role in delivering valuable insights. This concerted effort would considerably advance the comprehension and applicability of outcomes, particularly within the medical field.

In the setting of OLMs, radiomics faces a unique challenge since the area of interest, the occult lymph node, either does not appear in the image (in PET) or has limited volume (in CT). In the future, the utilisation of deep learning techniques has the potential to facilitate comprehensive imaging evaluation and automate the extraction of information from both the primary tumour and the lymph node chain, eliminating the need for manual delineations, given sufficient training data.

### 4.3. Conclusions

In conclusion, the prediction of OLMs remains an unmet clinical need as it is essential for planning the appropriate surgical approach, preserving lung function, and enabling accurate prognostication. The existing models exhibit suboptimal performance, underscoring the urgent requirement to explore and implement novel tools. Radiomics, whether based on machine learning or deep learning, has the potential to enhance the current models, resulting in improved accuracy. Therefore, adopting an interdisciplinary approach seems the most promising strategy for addressing the challenge of predicting OLMs in patients affected by lung cancer.

## Figures and Tables

**Table 1:** Clinical characteristics of the selected studies. SD: standard deviation; M: male; F: female; R: retrospective; [^18^F]FDG: [^18^F]fluorodeoxyglucose; ADC: adenocarcinoma; SCC: squamous cell carcinoma; LLL: left lower lobe; LUL: left upper lobe; RLL: right lower lobe; RML: right middle lobe; RUL: right upper lobe; T: tumour; N: node; cT: clinical tumour stage; pN: pathological nodal stage; N/A: not available. The number of patients involved as a validation cohort is reported in the brackets.

Authors [Ref.]	Year	Number of Patients	Mean Age ± SD (Testing Set)	Gender (M:F)	Smoker/ Non-Smoker	Design	Imaging Modality	Reference Standard	Histology	Tumour Location	Clinical T Stage	Mean T Diameter * ± SD (mm)	OLMs (%)/Non-OLMs (%)	Pathological N Status
**Zhong et al. [48]**	2018	492	61.4 ± 9.7	173:319	363/129	R	Unenhanced CT	Histology	ADC	Upper = 270 Middle and lower = 222	cT1-3	32.7 ± 15.2	78 (16)/414(84)	N/A
**Zhang et al. [7]**	2021	160 (84)	N/A	106:138	71/173	R	Contrast-enhanced CT	Histology	ADC	Upper = 137 Middle and lower = 107	cT1-2	30.4 ± 9.3	55(23)/189(77)	N/A
**Wang et al. [3]**	2021	236 (134)	62.95 ± 9.4	205:165	N/A	R	[^18^F]FDG PET/CT	Histology	ADC	LLL = 57 LUL = 95 RLL = 85 RML = 27 RUL = 106	N/A	28.23 ± 10.26	98(26)/272(74)	pN0 = 272 pN1 = 46 pN2 = 52
**Qiao et al. [49]**	2022	159 (69)	N/A	113:115	97/131	R	[^18^F]FDG PET/CT	Histology	ADC SCC	LLL = 43 LUL = 55 RLL = 51 RML = 11 RUL = 68	N/A	32.0 [2.3–4.4] ^†^	85(37)/143(63)	N/A
**Ouyang et al. [50]**	2022	376 (58)	N/A	193:241	104/330	R	[^18^F]FDG PET/CT	Histology	ADC	LLL = 66 LUL = 113 RLL = 91 RML = 34 RUL = 130	N/A	23.31 ± 10.36	91(21)/343(79)	pN0 = 343

* In cases with separate data for training and validation sets, we reported the data related to patients with OLMs included in the training set; ^†^ median tumour long diameter (interquartile range).

**Table 2:** Summary of methodologies and results of the selected articles. SENS: sensitivity; SPEC = specificity; PPV: positive predictive value; NPV: negative predictive value; RQS: radiomic quality score; ACC: accuracy; AUC: area under the receiver operating characteristic curve; LASSO: least absolute shrinkage and selection operator; CNN: convolutional neural network; ICC: intraclass correlation coefficient; OLM: occult lymph node metastasis; SVM: support vector machine; CT: computed tomography; PET: positron emission tomography.

Authors	Segmentation	Feature Extraction	Methods	Results	SENS * (%)	SPEC * (%)	PPV * (%)	NPV * (%)	ACC * (%)	RQS
**Zhong et al.,** **2018 [48]**	Manual segmentation of primary tumour using CT	300 radiomic features (first-order and texture features) Wavelet-based filtering	Feature reduction based on inter-reader reproducibility (assessed through the ICC) SVM classifier to derive a radiomic score Multivariate logistic regression to predict mediastinal LN metastasis from clinical features versus the radiomic score	Radiomic signature showed better predictive results compared to the clinical histopathological model (cross-validated SVM AUC = 0.97)	94.8 ^§^	92 ^§^	N/A	N/A	N/A	36%
**Zhang et al.,** **2021 [7]**	Manual segmentation of primary tumour using CT and multiple reader segmentations for 30 patients	851 radiomic features (shape, first-order, and texture features) Wavelet-based filtering	Feature reduction based on inter-reader reproducibility (assessed through the ICC) LASSO for radiomic feature selection and to derive a radiomic score Logistic regression with backward stepwise selection to integrate clinical features and radiomic score	Creation of a nomogram incorporating clinical and radiomic features (C-index = 0.81 in the validation cohort)	76.5 ^‡^	64.3 ^‡^	36.6 ^‡^	91.0 ^‡^	N/A	42%
**Wang et al.,** **2021 [3]**	Manual segmentation of the primary tumour in PET images and double reader segmentations for 60 patients	107 radiomic features (shape, first-order, and texture features)	Feature reduction based on inter- and intra-reader reproducibility (assessed through the ICC) LASSO for radiomic feature selection and to derive a radiomic score Logistic regression with backward stepwise selection to integrate clinical features and radiomic score	Creation of a nomogram incorporating clinical and radiomic features (C-index = 0.77 in the validation cohort)	N/A	N/A	N/A	N/A	N/A	39%
**Qiao et al.,** **2022 [49]**	Tumour segmentation in both CT and PET images using both manual and semi-automatic methods	1316 radiomic features (shape, first-order, and texture features) Local binary pattern, Laplacian of Gaussian, and wavelet-based filtering	Feature selection using LASSO and extremely randomised trees Multivariate logistic regression to derive a radiomic score Logistic regression with backward stepwise selection to integrate clinical features and radiomic score	Creation of a PET/CT-based nomogram including clinical and radiomic features (AUC = 0.88 on the test set)	72.9 ^◊^	87 ^◊^	76.8 ^◊^	84.5 ^◊^	81.8 ^◊^	39%
**Ouyang et al.,** **2022 [50]**	Segmentations were not performed; axial, coronal, and transversal 2D slices were used	Deep-features of the CNN	Development of a CNN model (Inception v3) to predict OLM from PET, CT, and PET and CT images	The model based on both PET and CT imaging achieved the best performances (AUC = 0.87 in the prospective test set)	87.5 ^◊^	80 ^◊^	N/A	N/A	81 ^◊^	13% ^†^

* In cases with separate data for training and validation sets, we reported the data related to patients with OLMs included in the training set; ^§^ values referring to total radiomic signature; ^‡^ values referring to radiomic score; ^◊^ values referring to the performance of the predictive model combining PET + CT; ^†^ estimation performed using the RQS 2.0 metric, which is currently under development for use with deep learning studies specifically (https://www.radiomics.world/rqs2/dl accessed on 14 April 2023).

## Data Availability

All necessary data are contained within the text.
